# UV-A Irradiation Increases Scytonemin Biosynthesis in Cyanobacteria Inhabiting Halites at Salar Grande, Atacama Desert

**DOI:** 10.3390/microorganisms8111690

**Published:** 2020-10-30

**Authors:** Gabriela Orellana, Benito Gómez-Silva, Milton Urrutia, Alexandra Galetović

**Affiliations:** 1 Laboratorio de Bioquímica, Depto. Biomédico, Facultad Ciencias de la Salud and Centre for Biotechnology and Bioengineering (CeBiB), Universidad de Antofagasta, Avenida Angamos N° 601, Antofagasta 12701300, Chile; gorellanagonzalez@gmail.com (G.O.); benito.gomez@uantof.cl (B.G.-S.); 2 Universidad de Antofagasta, Avenida Angamos N° 601, Antofagasta 12701300, Chile; milton.urrutia@uantof.cl

**Keywords:** scytonemin, cyanobacteria, UV-A radiation, *Halothece*, halite, Atacama Desert

## Abstract

Microbial consortia inhabiting evaporitic salt nodules at the Atacama Desert are dominated by unculturable cyanobacteria from the genus *Halothece*. Halite nodules provide transparency to photosynthetically active radiation and diminish photochemically damaging UV light. Atacama cyanobacteria synthesize scytonemin, a heterocyclic dimer, lipid soluble, UV-filtering pigment (in vivo absorption maximum at 370 nm) that accumulates at the extracellular sheath. Our goal was to demonstrate if UV-A irradiations modulate scytonemin biosynthesis in ground halites containing uncultured *Halothece* sp. cyanobacteria. Pulverized halite nodules with endolithic colonization were incubated under continuous UV-A radiation (3.6 W/m^2^) for 96 h, at 67% relative humidity, mimicking their natural habitat. Scytonemin content and relative transcription levels of *scy*B gene (a key gene in the biosynthesis of scytonemin) were evaluated by spectrophotometry and quantitative RT-PCR, respectively. After 48 h under these experimental conditions, the ratio scytonemin/chlorophyll *a* and the transcription of scyB gene increased to a maximal 1.7-fold value. Therefore, endolithic *Halothece* cyanobacteria in halites are metabolically active and UV radiation is an environmental stressor with a positive influence on *scy*B gene transcription and scytonemin biosynthesis. Endolithobiontic cyanobacteria in Atacama show a resilient evolutive and adaptive strategy to survive in one of the most extreme environments on Earth.

## 1. Introduction

The Atacama Desert is considered one of the driest and oldest extreme environments under permanent desiccation, with one of the highest levels of solar radiation on Earth [1,2,3]. This coastal nonpolar desert has unique geomorphological features that include longitudinal mountain ranges, salt-rich basins and a central hyperarid depression; all of these features provide diverse habitats with microbiota under continuous scrutiny [4,5,6,7,8].

**Figure 1:**
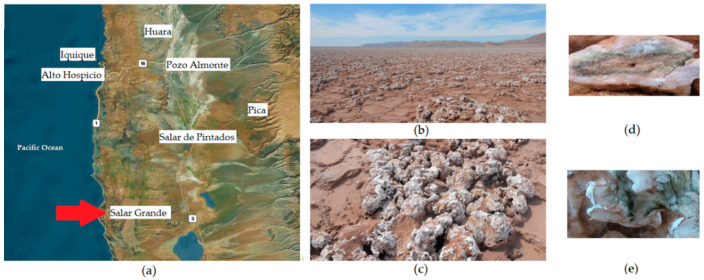
Halites in Salar Grande. (**a**) Localization map of Salar Grande in northern Chile (source: https://satellites.pro/mapa_de_Chile#-20.564653,-69.455566,9), with the arrow indicating the location of Salar Grande; (**b**) General view of the sampling site at Salar Grande; (**c**) Close up on halite pinnacles at Salar Grande; (**d**) Broken halite showing endolithic colonization (green line); (**e**) Halite showing epilithic colonization.

Among the various salt pans in Atacama, Salar Grande is a non-fossil coastal basin located at the eastern slopes of the Coastal Range, at the Tarapacá Region in northern Chile (Figure 1a,b) [9,10]. This basin, rich in evaporitic rocks, has an annual mean temperature of 20 °C, and a relative humidity (RH) and UV irradiation close to 50% and 550 µmol s^−1^ m^2^, respectively [4]. Its surface is covered with flat salt-plates full of upgrowing halite nodules, containing 95–99% NaCl, whose origin has been established as dating back to millions of years ago [9,10,11].

Cyanobacteria-dependent microbial communities inhabit halites and include members of the three domains of life [4,11,12]. Permanent desiccation in Salar Grande is a consequence of the high solar insolation and absence of rains. Evaporitic NaCl-rich rocks are appropriate habitats for microbial colonization, since they provide conditions for harvesting liquid water by deposition of fog droplets on the surface of halites and from atmospheric water vapor by NaCl deliquescence [13,14]. NaCl deliquescence starts when the relative humidity reaches 75% and atmospheric water is trapped by the salt crystals, forming saline brines within the micropores in the halites [15] (Figure 1c).

Metagenomics analyses on the microbial communities colonizing halites at Salar Grande have shown that a unicellular cyanobacterium species of the genus *Halothece* is a dominant member, among other phototrophic microorganisms [4,11,12]. This cyanobacterium performs oxygenic photosynthesis within the translucent porous halites during periods when liquid water and solar radiation are available and the lithobiontic community is partially protected from the incident UV radiation [14,15]. Members of the genus *Halothece* have been described as halotolerant, moderate thermophiles that reproduce by binary fission, are able to grow at 45 °C, and may inhabit hypersaline environments [16].

Microorganisms adapt to extreme environmental conditions by synthesizing secondary metabolites [17], such as exopolysaccharides, sun-screening molecules (mycosporine-like amino acids and scytonemin), antioxidants (carotenoids), and osmoregulatory molecules [7,13,18]. Scytonemin is a chemically stable, indole-alkaloid dimeric molecule that is only synthesized by some cyanobacterial genera, and it was first identified as a yellow-brown pigment on the cyanobacterial sheath [19]. This pigment is a UV-A protecting molecule with absorption maxima at 370 nm and 384 nm in vivo and in organic solvents, respectively. Its yellow reduced or brown oxidized forms (546 and 544 g/mol, respectively) reach nearly 5% *w/w* in cultured cyanobacteria [20,21]. Also, the existence of the scytonemin derivatives tetramethoxyscytonemin, dimethoxyscytonemin, and scytonine has previously been reported in the organic extract of *Scytonema* sp. [22]. Scytonemin biosynthesis is stimulated by stressors such as UV-A irradiation, salinity, and desiccation [23], while tyrosine and tryptophan are precursors in its biosynthesis [24]. Epilithic cyanobacteria have a scytonemin content 12-fold higher than their endolithic counterparts in Atacama halites under natural solar irradiation [18].

Soule et al. (2007) [25] identified 18 genes in *Nostoc punctiforme* ATCC 29133 encoding for enzymes involved in scytonemin, aromatic amino acids, and regulatory proteins biosynthesis. Eight genes were directly involved in the pigment synthesis, six were annotated as the scytonemin genes (*scy*A to *scy*F), and two genes, *tyr* and *trp*, were necessary for products involved in the formation of aromatic amino acids and other intermediary metabolites. Furthermore, the products of two other genes, *aro*G and *aro*B, were involved in the synthesis of shikimic acid [26], a metabolite needed for the synthesis of aromatic amino acids and other aromatic compounds [27]. In cyanobacteria, the cytoplasmic enzyme coded by the *scy*B gene transforms tryptophan into indole-3-pyruvate; then, the first committed intermediate for scytonemin synthesis is formed by a leucine dehydrogenase encoded by the *scy*A gene. The first indole monomer is made after indole-3-pyruvate plus p-hydroxyphenylpyruvate condensation. The monomer is then transported to the periplasm by the protein encoded by the *scy*C gene. At the periplasm, a second chemical modification (indole reaction) forms a second alkaloid-like monomer, and oxidized scytonemin is generated after the monomer’s condensation. Oxidized scytonemin transport to the extracellular space is mediated by enzymes with transmembrane domains encoded by the *scy*D, *scy*E, and *scy*F genes. Chemically reduced scytonemin is then formed in the cyanobacterial outer layers [28].

The presence or absence of metabolic activity in lithobiontic microbial consortia has been an open question since their presence was reported in Atacama halites and other lithic habitats [29,30]. Although there are limited windows of time for oxygenic photosynthesis, this central metabolic pathway has been demonstrated to occur in halites by direct and indirect measurements [31].

Members of the microbial consortia in lithic habitats have adaptive strategies to cope with the high solar UV radiation in Atacama [13]; among them, scytonemin is an extracellular-located pigment that diminishes photodamage [13,18]. Considering that UV-A irradiation increases scytonemin content in cultured cyanobacteria [32,33], we hypothesized that the biosynthesis of scytonemin is positively influenced by UV-A light in uncultured cyanobacteria inhabiting halite nodules. Here, we demonstrate for the first time that uncultured endolithic *Halothece* cyanobacteria under low relative humidity are metabolically active and show an increment in *scy*B gene transcription and scytonemin biosynthesis during exposure to UV-A irradiation. Thus, the evolution of life has provided the appropriate primary producers to colonize and sustain microbial communities in one of the most polyextreme habitats in our planet.

## 2. Material and Methods

### 2.1. Sampling and Handling of Halites

Halite sampling was conducted at Salar Grande (2056.310′ S, 70.00.319° W) (Figure 1a–c) during May 2014, December 2015, and July 2016. Samples (300 g) of colonized halites with evident brown-green epilithic biofilms were collected in sterile plastic bags (Nasco Whirl-Pak, Fort Atkinson, WI, USA) under aseptic conditions and stored until their use. Collected halites were broken under sterile conditions in a biosafety cabinet to access to inner endolithic colonization (green biofilm) (Figure 1d,e), which were scrapped, and the halite powder was collected and used to evaluate the effect of artificial UV-A irradiation on scytonemin content and gene expression.

### 2.2. Experimental Setup

Irradiation experiments were conducted within a closed wooden cabinet to exclude external light, and supplied with a combination of two white fluorescent and three ultraviolet light lamps (UV-A lamps: actinic BL special model TL- D15W; fluorescent lamps: T8 VKB, 18 W) located at its inner top. Temperature and RH within the experimental cabinet were registered by an autonomous HOBO Prov2 sensor, and the UV-A light meter MU-100 was used to measure the UV-A radiation at the samples level. Halites samples were maintained in a humidity chamber (67% RH, 25 °C) for at least one week before irradiation. To guarantee representative halite samples, a grid sampling method was applied [34]. A 24-well plate (NUNC, BIO SIGMA, Aldrich, Dutscher Group, Brumath, France) was divided into three 8-well quadrants, and pulverized halites (0.3 g) were randomly distributed into each quadrant as mono-granular layers. UV-A irradiation experiments in tetraplicates were conducted for 96 h under continuous white light (1.2 W/m^2^) plus UV-A light (3.6 W/m^2^). Irradiated halites were retrieved every 24 h into sterile tubes. Halites irradiated with white light, in absence of UV-A radiation, were used as controls.

### 2.3. Scytonemin and Chlorophyll Content

Pulverized halites (about 1.2 g) from UV-A and non-UV-A irradiated samples were transferred to 15-mL tubes and extracted overnight with 3 mL of 100% acetone, at 4 °C, in darkness. The acetone extracts were centrifuged at 2400× *g* for 4 min at room temperature, in a table-top centrifuge IEC center CL2. The supernatants were collected, and the corresponding 300–700 nm absorption spectra were obtained in a spectrophotometer Shimadzu UV-1601. Scytonemin and chlorophyll contents were calculated from the corresponding absorbances at 384 nm (scytonemin) and 663 nm (chlorophyll *a*) using the extinction coefficients in acetone of 92.6 and 112.6 g^−1^ L cm^−1^ for chlorophyll *a*, respectively [35]. The collected data was analyzed using a box plot and the R statistical program [36], and minimum and maximum values, median, and first and second quartiles were grouped. The results were expressed as the content ratio of scytonemin to chlorophyll versus time of exposure to UV-A light.

### 2.4. RNA Extraction and cDNA Synthesis

Irradiated halite samples (~2 g) were dissolved in a 20% *w/v* NaCl solution prepared with diethylpyrocarbonate DEPC-treated autoclaved water, and cells were recovered by centrifugation (10,000 × *g*, 15 min), at room temperature. Total RNA was extracted from the cell pellet with the Power Soil RNA Isolation kit (MoBio Laboratories Inc., Solana Beach, CA, USA) following the manufacturer’s instructions, and incubated with RNAse-free DNase, during 30 min at 37 °C. Total DNase-treated RNA was the template for cDNA synthesis using the TM kit Thermo Script RT-PCR System (Invitrogen, Life Technologies, Carlsbad, CA, USA), following the manufacturer’s instructions, and cDNA was stored at −20 °C.

### 2.5. Genes and Primers for PCR Amplifications

*scy*B gene was amplified using the primers 5′GCCTGCTCCCATCACATCATTAG3′ and 5′TGCGCCAAAAAGCCGTT3′ to obtain amplicons of 220 bp [32]. Ribosomal 16S RNA gene was amplified using the cyanobacterial specific primers CYA106F and CYA781Ra plus CYA781Rb, in order to obtain fragments of 800 bp [37], and primers designed from the *Nostoc* sp 7120 genome. Primers 16SHalotheF and 16SHalotheR were used to amplify a 209 bp fragment [38]. The *rnp*B gene fragment of 243 bp was amplified with primers *rnp*BF and *rnp*BR [32].

### 2.6. Cloning and Sequencing of 16S rRNA and scyB Genes

PCR products were cloned into the pGEM-T easy vector (Promega Corporation, Madison, WI, USA) and transformed into *Escherichia coli* strain DH-5α. Plasmids with inserts closer to the expected 200 bp size were sequenced in Macrogen, Korea ((http://ADN.macrogen.com/), using the universal primers M13F (5′GTAAAACGACGGCCAGT3′) and M13R-pUC (5′ CAGGAAACAGCTATGAC3′).

### 2.7. Similarity Searches for Nucleotide Sequences of scyB and 16S rRNA Genes

Searches were done by BLAST [39] in NCBI-Gene Bank (http://www.ncbi.nlm.nih.gov/GenBank), using the blastn bioinformatics tool version 2.2.29 (http://blast.ncbi.nlm.nih.gov/Blast.cgi). Only alignments with an E-value between 0 and 1 × 10^−3^ were considered.

### 2.8. Quantitative Real Time PCR

The *scy*B gene was amplified by qPCR using the specific primers and protocol described by Soule et al. (2009 b) [32]. The *rnp*B gene, as a housekeeping gene, was used for amplification, since its transcription is not affected by UV light [40]. qRT-PCR reactions were performed using 1.0 ng of cDNA as template, and primers at 200 nM, in a final volume of 20 µL of SYBR Green Supermix with Rox (Bio Rad Laboratories, Inc, Redmond, WA, USA). Each amplicon was about 200 bp in length. The annealing temperature for *scy*B and *rnp*B genes was 55 °C and 53 °C, respectively. The concentration of primers for *scy*B and *rnp*B genes were 250 nM/50 nM and the 250 nM/100 nM, respectively. The comparative transcripts levels were determined after normalization with *rnp*B amplicons. The experiments were performed in triplicate.

### 2.9. Validation of Results by the ∆∆CT Method

The ∆∆CT method was used to determine if the *rnp*B and 16S rRNA reference genes have the same PCR efficiency [41,42]. The arithmetic formula to validate the results is as follows:

2^−∆∆CT^ (1)

The ∆∆CT is given by (CT sample − CT reference gene). Then, the standard deviation (S) of the ∆CT value is calculated, using the following formula, where S_1_: target (*scy*B gene) and S_2:_ reference (*rnp*B gene):

S = (S_1_^2^ + S_2_^2^)^1/2^ (2)

Then we proceed using the following formula to obtain the value of −∆∆CT:

∆∆CT + s = −∆∆CT(3)

The value of −∆∆CT, when used in the formula already defined above, makes it possible to calculate the number of times an increase or decrease in the relative expression of the *scy*B gene occurs.

### 2.10. Statistical Treatment

Exploratory analysis of the amount of scytonemin was performed using the box plot by comparing the groups according to hours of exposure. The amounts obtained were compared using the Tukey test at a 95% confidence level. The R software program (R Core Team, Vienna, Austria, 2017) [43] was used to carry out the analyzes.

## 3. Results and Discussion

### 3.1. Quantification of Scytonemin

Previous reports on the Atacama lithobiontic microbial communities inhabiting halite nodules showed that rain events are practically absent in this hyperdesert; however, resilient microbial assemblies can overcome the permanent desiccation by obtaining liquid water from regular fog events and NaCl-dependent deliquescence when at least 75% RH is reached within the halites [12,13]. Atacama is also under one of the highest levels of UV radiation in our planet, and microbial assemblies colonizing halites use scytonemin as a key UV-protecting pigment [18,44]. Our research objective was to test the effect of UV-A irradiations on scytonemin biosynthesis in endolithic cyanobacteria colonizing halites, mimicking the regular environmental conditions.

There is no solid information available on the level of UV-A radiation reaching the endolithic micro-environment in halites at Salar Grande, Atacama, which is protected by just a few millimeters of salt layers below the sun-exposed rock surface. In this context, solar irradiation in Salar Grande is close to 120–500 Wm^−2^ [4]; also, Dávila et al. (2015) [31] reported that the metabolic activity was centered in the endolithic communities of halites, and only 0.02% of incident photosynthetically active radiation PAR light on the halite surface reached the endolithic environment. With these facts in mind, we decided to use a UV-A irradiation dose of 3.6 Wm^−2^, which was lower than those used (5–10 Wm^−2^) to evaluate the effect of UV-A on scytonemin content and gene expression in cultured *Noctoc* cyanobacterium [26,32].

**Figure 2:**
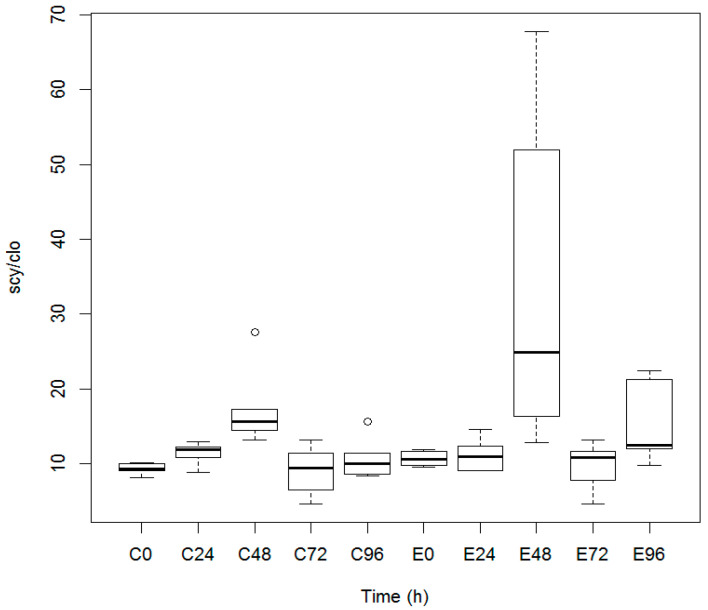
Effect of UV-A irradiation on the scytonemin content in cyanobacteria contained in Atacama halites. Finely grained halites were continuously irradiated with 3.6 W/m^2^ of UV-A plus 1.2 W/m^2^ white fluorescent light for four days. Samples (E.0 to E.96) were retrieved at four 24-h intervals and extracted with acetone to evaluate the scytonemin and chlorophyll *a* content, expressed as a scy/Chl *a* ratio. Results were compared with those from controls (C.0 to C96) incubated under similar conditions with only white fluorescent light. The Tukey test for multiple comparisons of means was applied (*p* = 0.05) to affirm that the mean of the scytonemin of the group E48 was different when compared with the control and other experimental groups. Outliers points are marked with a circle. The experiment was done in triplicate.

The UV-A-protecting pigment content in lithobiontic cyanobacteria from pulverized halites was normalized as a ratio scytonemin/chlorophyll *a* (scy/chl *a*) to evaluate changes of the pigment concentration during the experiments. Desiccated halite samples were incubated under constant UV-A irradiation (3.6 W/m^2^) and at a suboptimal 67% RH atmosphere. Samples were retrieved after 24 h, 48 h, and 96 h of incubation to obtain their UV-VIS absorption spectra, to quantitate pigment contents, and to compare those with the experimental controls. A slight increase in the ratio of scy/chl *a* was observed after the 24-h incubation, followed by a larger ratio increase (1.7-fold) after the 48-h incubation, in comparison with the controls (Figure 2). Longer incubation times (72–96 h) rendered a decrease in the scy/chl *a* ratio. Therefore, lithobiontic *Halothece* members of microbial communities colonizing halites in Atacama were metabolically active and synthesized scytonemin after exposure to UV-A light (acting as an inducer of scytonemin biosynthesis) under the laboratory conditions used in this work.

Comparatively, a 4-fold increase in scytonemin was reported in *Nostoc punctiforme* ATCC 29133 after 48-h incubations under continuous UV-A exposure (5 W/m^2^) supplemented with white fluorescent light (10 W/m^2^), in comparison with white light control [32]. Also, the cyanobacteria *Nostoc commune* irradiated for 2.5 days with UV-A (1.7 W/m^2^) showed a 2–3 times increase in scytonemin production in comparison with cultures treated with UV-B light [45].

All the acetone extracts from the UV-A irradiated Atacama halites showed the characteristic brown coloration of scytonemin; however, the absorption at 384 nm decreased at incubations longer than 48 h. Although scytonemin is a stable pigment, it is known that UV-A light generates modifications in the metabolism of cyanobacteria, triggering a series of physiological and molecular changes that affect the synthesis of the pigment [46]. Since UV-A radiation produces oxidative damage due to the generation of reactive oxygen species (ROS) [47], this could cause degradation or metabolic modification of scytonemin, as suggested by Fleming and Castenholz (2007) [48]. In fact, Bultel-Poncé, V.; et al. (2010) [22] found three new derivatives of scytonemin in *Scytonema* sp., denominated tetramethoxiscytonemin (purple; absorption peaks at 212 and 562 nm), dimethoxyscytonemin (dark red; absorption peaks at 215, 316, and 422 nm), and scytonin (brown; absorption peaks at 207, 225, and 270 nm). The acetone extract from irradiated halite samples had a brown coloring characteristic of scytonemin, and the corresponding spectra at the visible region did not show absorbance maxima at 562 or 422 nm, discarding the presence of tetramethoxiscytonemin or dimethoxyscytonemin. Therefore, in spite of the high photostability of the scytonemin, it is presumed that as part of the photoprotective action and/or due to the exposure to UV light and reactive species of oxygen in UV-A irradiated Atacama halites, the scytonemin could suffer chemical modifications and derivations of scytonin-like or other unknown molecules. Then, the diminished absorbance at 384 nm, may be partially explained by changes in the scytonemin structure and absorption maxima.

In this context, and based on Raman spectroscopy, Varnali and Edwards (2014) [49] proposed the existence of stable reduced and oxidized forms of scytonemin in stressed cyanobacteria. These modified scytonemin compounds have absorption characteristics that are different from scytonemin.

On the other hand, it is known that tryptophan is an important precursor in scytonemin synthesis, and the expression of tryptophan genes is activated and increased in response to UV-A light [32]. Long exposures to UV-A light and concurrent scytonemin synthesis increase the demand for free tryptophan; therefore, this possibility could trigger a decrease in the pigment synthesis due to depletion in the cell reservoir of this amino acid after 72–96 h of incubation.

Another interesting point to mention is desiccation. Cyanobacteria inhabiting halites in the Atacama Desert are subjected to desiccation, mean daily temperatures of 20–24 °C, and 51.45% mean RH [10]. Also, when RH reaches or surpasses 75%, the salt rocks experience deliquescence, allowing the community of microorganisms to collect liquid water from atmospheric water vapor. In this study, the in vitro experiments were carried out at a RH value lower than that needed for NaCl deliquescence; therefore, the cyanobacteria were in a permanent condition of desiccation during the experiment. Maximum scytonemin content was observed under desiccating conditions (67% RH) at 48 h of continuous irradiation with UV-A light; concurrently, control samples showed a slight increase at the same period but without UV-A irradiation (see the ratio of scy/chl *a* in Figure 2). Our work in Atacama halites showed that desiccation was not causative by itself for increasing the synthesis of scytonemin in irradiated ground Atacama halites.

These results agree with the data in the literature showing that pigment synthesis is mainly induced by UV-A radiation and to a lesser extent by environmental factors such as temperature and salinity [47,48]. Mishra et al. (2015) [50] reported that *Chroococcidiopsis* and *Nostoc punctiforme* produce more scytonemin when they are irradiated with UV-A light and submitted to cycles of desiccation–hydration. Therefore, protection provided by scytonemin is highly relevant during desiccating periods, since other metabolic processes are inactivated due to the lack of enough hydration. In fact, scytonemin in *Nostoc punctiforme* is produced during several months of continuous exposure to UV-A light. Also, a UV-A dependent scytonemin biosynthesis under natural conditions has been previously reported in *Calothrix* sp. [35].

### 3.2. Amplification of scyB Gene

**Figure 3:**
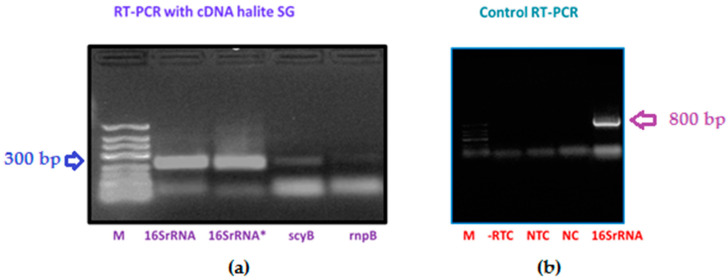
Amplification of *scy*B, *rnp*B, and 16S rRNA. Samples of cDNA from halites were amplified by RT-PCR. Panel (**a**): 16SrRNA (191 bp); 16SrRNA* (16S rRNA gene from *Halothece* sp) [38]; scyB (220 bp amplicon); and rnpB (253 bp amplicon) [32]. Panel (**b**): -RTC (reverse transcriptase control, total RNA without reverse transcriptase); NTC (non-template control); NC (water, negative control); and positive control 16S rRNA (800 bp amplicon from cDNA) with 106F and reverse primer CYA781R was an equimolar mixture of CYA781R(a) and CYA781R(b) [37]. M: low range 700–50 bp DNA marker.

The amplification of *scy*B and *rnp*B genes was performed in cDNA prepared from total RNA extracted from UV-A irradiated halite samples. The sizes of the amplified fragments, including the positive control 16S rRNA, agreed with those reported in the literature [32] (Figure 3). Our results showed that the expression of the *scy*B gene is activated by UV-A light in the unculturable *Halothece* cyanobacterium inhabiting halite nodules. The *scy*B gene encodes for the enzyme tryptophan dehydrogenase involved in the formation of indole 3-pyruvate, a metabolic intermediate in the scytonemin biosynthetic pathway [51].

### 3.3. Relative Expression of the scyB Gene in Cyanobacterial Inhabiting Halites by qRT-PCR

**Figure 4:**
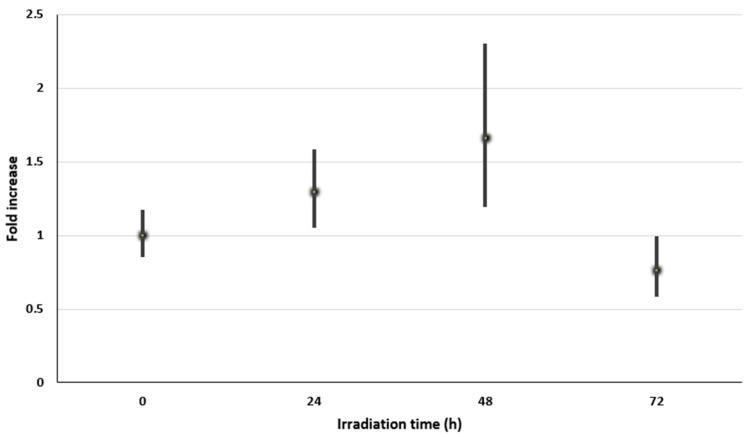
Relative expression of *scy*B gene in ground halites irradiated with UV-A light. Finely grained halites were continuously irradiated with 3.6 W/m^2^ of UV-A plus 1.2 W/m^2^ white fluorescent light for 72 h. Samples were retrieved at selected intervals, and total RNA was extracted to prepare cDNA. Experiments were conducted in triplicate.

The relative expression of the *scy*B gene was performed by qRT-PCR and ∆∆CT method. The technique requires the use of an endogenous control to visualize the transcript levels. The *rnp*B gene was the control selected for this work, since it is involved in RNA synthesis and its transcript level does not vary during exposure to UV-A light [32]. A 1.7-fold increase in the relative expression of the *scy*B gene, with respect to the control without UV-A irradiation (Table 1 and Figure 4), was observed after 48 h with continuous UV-A irradiation.

UV-A irradiation triggered a nearly 2-fold increase in *scy*B gene expression in cyanobacteria-dominated Atacama halites. Comparatively, cultured *Nostoc punctiforme* ATCC 29133 under UV-A plus white light irradiation showed a 3- to 5-fold increase in *scy*B gene transcription in comparison with the control cells incubated only with white light [25]. Relatively low levels of gene transcription are expected for genes involved in pigment synthesis under UV-A light in comparison with genes involved in central metabolic pathways [32]. In fact, Soule et al. (2009b) [32] showed that after 48 h of UV-A irradiation, maximal levels of gene transcription (*scy*A to *scy*F) were reached for proteins involved in the biosynthesis of scytonemin as well as for housekeeping genes for tryptophan and aromatic amino acids biosynthesis. In concordance with our results of maximal levels of transcription of *scy*B gene in UV-A-irradiated Atacama halites, it can be inferred that scytonemin synthesis is dependent upon the appropriate tryptophan pool of cyanobacteria cells being exposed to UV-A light.

Laboratory-grown *Anabaena* PCC 7120 and *Lyngbya* PCC 8106 did not synthesize scytonemin in the absence of UV-A exposure, even though they have the gene clusters needed for its biosynthesis [32]. Comparatively, Atacama halite samples retrieved from their natural UV-A-irradiated habitat, showed both a basal scytonemin content and *scy*B gene transcripts at the beginning of the irradiation experiments (Figure 4). During the 48-h incubation under UV-A light, the level of *scy*B gene transcription increased, correlating positively with a maximum increase in scytonemin content (Figure 2). Additionally, the *scy*B gene transcription level and scytonemin content decreased after 72 h of UV-A irradiation, which coincides with the decrease in scytonemin content (Figure 2 and Figure 4); an observation that can be associated with impediment in the repair processes needed to maintain appropriate transcript levels. In this context, Williamson et al. (2017) [52] showed a decrease in transcript levels in fibroblast cell cultures when exposed to UV-A; however, the levels of transcripts returned to normal levels due to cellular repair processes.

## 4. Conclusions

Endolithic cyanobacteria are metabolically active under the natural environmental conditions prevailing at Salar Grande, Atacama Desert. This was demonstrated by the UV-induced scytonemin biosynthesis in exposed ground halites containing uncultured cyanobacteria (*Halothece* sp.). Both *scy*B gene expression and scytonemin content nearly doubled after 48 h of continuous irradiation with 3.6 W/m^2^ UV-A plus 1.2 W/m^2^ white fluorescent lights, at 67% RH. This work is the first study on scytonemin synthesis in non-cultivable cyanobacteria adapted to inhabit and support lithobiontic microbial communities in evaporitic NaCl rocks in Atacama, and probably elsewhere.

Because scytonemin content decreased after 72 h of incubation, we hypothesize that UV-A exposures longer than 48 h transform scytonemin into scytonin-like derivates or, alternatively, the intracellular concentration of tryptophan becomes a limiting factor for the pigment biosynthesis. We propose that future work should be focused on the identification of scytonemin derivates after UV-A radiation, on the transcriptomics of genes involved in tryptophan biosynthesis, and on the repair mechanisms of UV induced damages and their effect on the level of transcripts during UV-A exposure.

## Figures and Tables

**Table 1:** Relative expression of the *scy*B gene in halites exposed to UV-A irradiation. This experiment was performed in triplicate.

UV-A Irradiation Time (h)	*scy*B (Mean C_T_)	*rnp*B (Mean C_T_)	∆CT (*scy*B-*rnp*B)	∆∆ CT (*)	Fold Increase (**)
0	29.67	32.68	−2.51	0	1 (0.85–1.17)
24	29.24	32.12	−2.88	−0.37	1.29 (1.05–1.58)
48	29.49	32.73	−3.24	−0.73	1.66 (1.19–2.31)
72	29.84	31.96	−2.12	0.39	0.76 (0.58–0.99)

(*) ∆∆ CT (∆C_T_ irradiated−∆C_T_ no irradiated); (**): *scy*B expression relative to the untreated control.
